# Total Hip Lithiasis: A Rare Sequelae of Spilled Gallstones

**DOI:** 10.1155/2018/9706065

**Published:** 2018-06-14

**Authors:** Vineet Tyagi, Daniel H. Wiznia, Adrian K. Wyllie, Kristaps J. Keggi

**Affiliations:** Department of Orthopaedics and Rehabilitation, Yale University School of Medicine, 47 College Street, Second Floor, New Haven, CT 06510, USA

## Abstract

Laparoscopic cholecystectomy is a surgical treatment for acute cholecystitis or symptomatic cholelithiasis. One potential complication, the spillage of gallstones into the peritoneal cavity, can form a nidus for infection and may be associated with hepatic, retroperitoneal, thoracic, and abdominal wall abscesses. We report a case of a patient presenting with a right iliopsoas abscess and an infected right hip prosthesis status postlaparoscopic cholecystectomy. A CT demonstrated that the acetabular shell was overmedialized and perforated through the medial wall. The patient was taken to the operating room for explantation of components. A collection of gallstones was identified deep to the acetabulum during the explantation. The case highlights the importance of avoiding overmedialization of the acetabular component, which can provide a direct route for infection into the hip joint.

## 1. Introduction

Laparoscopic cholecystectomy (LC) is the procedure of choice for routine gallbladder removal and is among one of the most frequently performed surgical procedures in the United States. One common complication of the laparoscopic technique is the spillage of gallstones. It is estimated that approximately 30% of stones are spilled into the intraperitoneal space owing to perforation of the gallbladder during dissection [1, 2]. It is reported that gallstones are dropped by surgeons in an additional 7% of cholecystectomies, and studies suggest that 16–50% of dropped stones are unrecovered [3, 4]. It is well recognized that retained stones can become niduses for infection that may result in complications. In their review of complications from dropped gallstones from 1987 to 2005, Zehetner et al. demonstrated that the most common complications were abscesses, fistulas, and sinus tracts, many of which can take weeks to years to present [2].

An iliopsoas abscess is a relatively uncommon condition, and diagnosis is often missed or delayed, resulting in subsequent increases in mortality and morbidity. An iliopsoas abscess is a purulent collection within the psoas muscle compartment [5]. The psoas muscle is a retroperitoneal muscle that originates from the lateral borders of the 12th thoracic to 5th lumbar vertebrae and inserts on the lesser trochanter of the femur [6]. The iliopsoas bursa is the largest bursa in the body and presents with several different anatomical variations. It may extend proximally into the iliac fossa and distally to the lesser trochanter. Communication between this bursa and the hip joint occurs in approximately 15% of adults. In patients with hip pathology, there is a 30–40% frequency of communication between the two structures [7]. Tracking infections from the retroperitoneal psoas to the hip region have been reported with cases of tuberculous vertebral disease. Infections may spread to adjacent tissues contiguous with the iliopsoas bursa and can potentially track into the hip joint. Dinç et al. reported a case series of 21 patients with tuberculous spondylitis who developed iliopsoas abscesses requiring percutaneous drainage [8]. These findings may also be seen in patients who have paraspinal abscesses secondary to tuberculosis, and drainage through a subinguinal approach may be necessary to evacuate the collection before it spreads to the hip joint [9, 10].

Prosthetic infection is a serious concern for all arthroplasty surgeons and can be a devastating complication. Excessive medialization of the acetabular component can lead to a perforation in the medial wall and a potential communication with the hip joint and intraperitoneal contents and can act as a potential track for infections. In the case report below, we describe how dropped gallstones seeded a hip periprosthetic infection because the acetabular component was overmedialized and breached the medial acetabulum cortex, which likely put the component in contact to the dropped gallstones.

Overmedialization or protrusio acetabuli can expose the metal surface of the acetabular prosthesis to pelvic organs such as the bladder, ureter, or colon which may cause serious injuries or infections. Grauer et al. reported a case of a bladder tear occurring intraoperatively during a revision total hip arthroplasty (THA) [11]. They postulated that previous hip procedures lead to adhesions of the bladder to the pelvic floor and could predispose the bladder to injury during THA revisions.

Furthermore, as more of these two stage revision procedures are performed, Huo et al. suggested that it may be useful to consider patient-specific, customized, temporary methyl methacrylate prosthesis used by the senior author since 1992 when performing two stage revisions to treat periprosthetic hip infections. These implants allow for improved femoral head offset and leg length symmetry and allow the patient to mobilize prior to their second stage revision [12, 13]. Here, we present the first reported case of spilled gallstones causing a periprosthetic total hip infection.

## 2. Case Report

**Figure 1:**
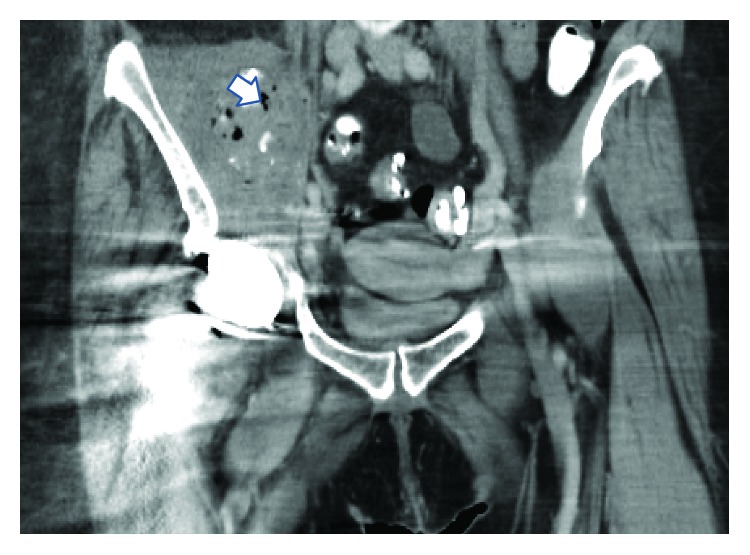
CT: right iliopsoas abscess with calcifications and air (arrowhead).

The patient was a 70-year-old female whose past medical history was significant for arthritis and a right total hip arthroplasty approximately 9 years ago. A laparoscopic cholecystectomy (LC) for acute cholecystitis was performed approximately at another hospital approximately two months prior to presentation. She developed a surgical site infection with *Escherichia coli (E. coli)* bacteremia following her LC and was treated successfully with intravenous (IV) antibiotics. The postoperative course was also complicated by choledocholithiasis requiring an endoscopic retrograde cholangiopancreatography (ERCP) with stone pulverization and placement of two plastic 10F × 12 cm biliary stents. Two days prior to admission, she was hospitalized at an outside facility in septic shock with fevers, chills, lethargy, altered mental status, and blood and urine cultures positive for *E. coli*. At that time, she endorsed right hip pain and an inability to move her hip or leg. A computed tomography (CT) scan of her right hip revealed two partly calcified soft tissue masses associated with the right iliopsoas and obturator internus muscles (Figure 1). A CT-guided fine needle biopsy of the right hip and psoas locules aspirated 100 mL of frank pus notable for a nucleated cell count of 344,000 (98% PMNs) with growth of *E. coli*. As a result, the patient was transferred to our institution with concerns for an iliopsoas abscess and a periprosthetic infection.

**Figure 2:**
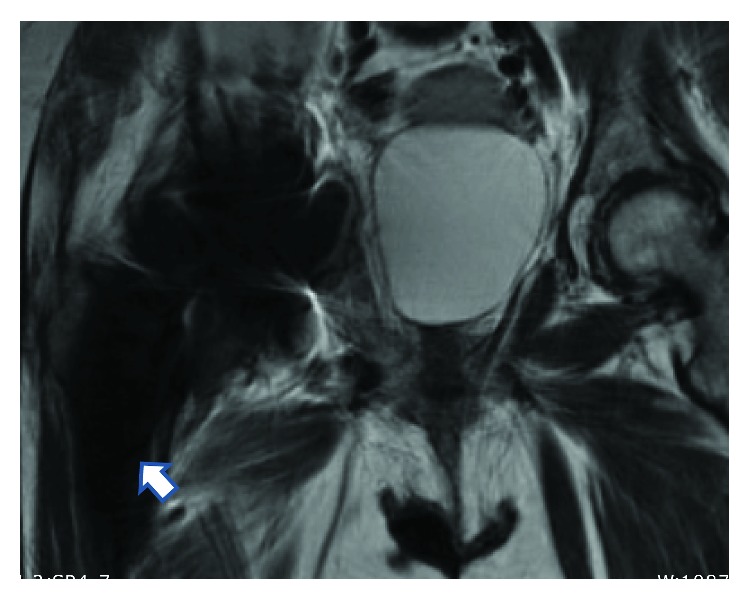
MRI right hip. Large air- and fluid-filled collection tracking along the iliopsoas bursa and psoas muscle (arrowhead).

**Figure 3:**
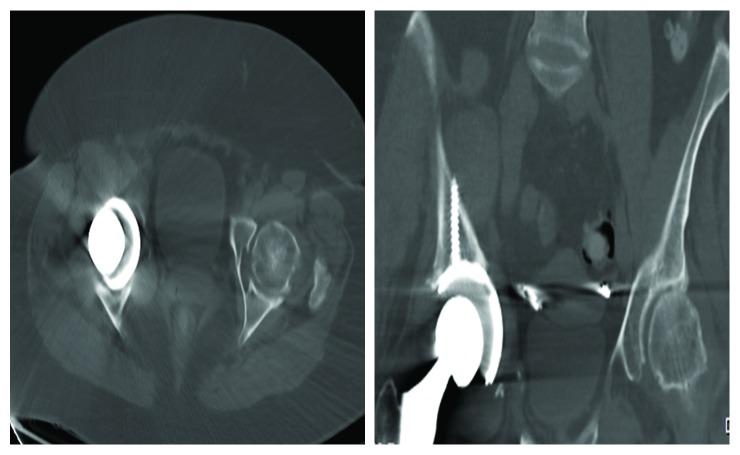
Axial and coronal CT scan showing excessive medialization of the acetabular component.

On admission, she was febrile to 102.7 F without any significant distress. Her physical examination was remarkable for a well-healed, right lateral hip incision with no erythema or drainage. She experienced pain with right hip flexion and internal rotation. Laboratory studies showed WBC, hemoglobin and hematocrit, basic metabolic profile, and liver function tests all within normal limits. A 3 cm hepatic abscess was identified on CT scan of the abdomen and pelvis. An MRI of the right hip showed a large air- and fluid-filled collection tracking along the iliopsoas bursa and psoas musculature into the pelvis (Figure 2). This collection communicated with the hip prosthesis, and on CT imaging, the acetabulum component appeared to be medialized beyond the medial wall of the acetabulum as depicted in Figure 3. Regarding the hepatic abscess, the patient was managed with IV antibiotics and interventional radiology (IR) placed drainage catheter.

To address the hip periprosthetic infection, the patient was managed in multiple surgical stages. In the first stage, an irrigation and debridement of the right hip and explantation of components were performed through an anterior approach. The femoral and acetabular components were explanted. Purulent material was seen draining from the pelvis through a medial acetabular wall defect into the hip joint. Approximately 1 liter of pus was evacuated from the hip joint. Multiple irregularly shaped granulated pea-sized pieces of hard brown substance were found deep in the acetabulum. A handful of this material was removed which suggested that these were spilled gallstones from the patient's recent LC.

**Figure 4:**
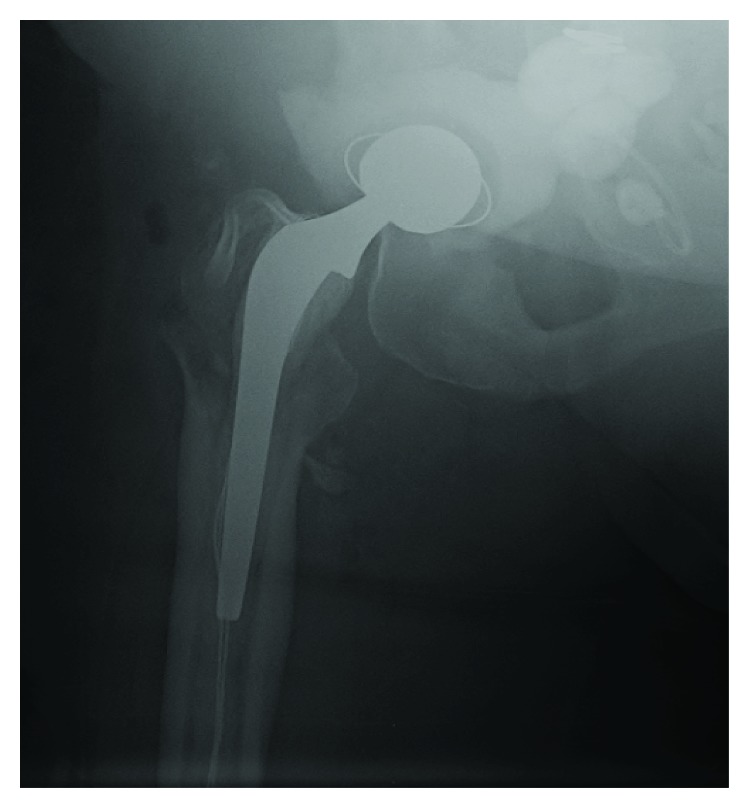
Stage 1: postoperative XR right hip.

**Figure 5:**
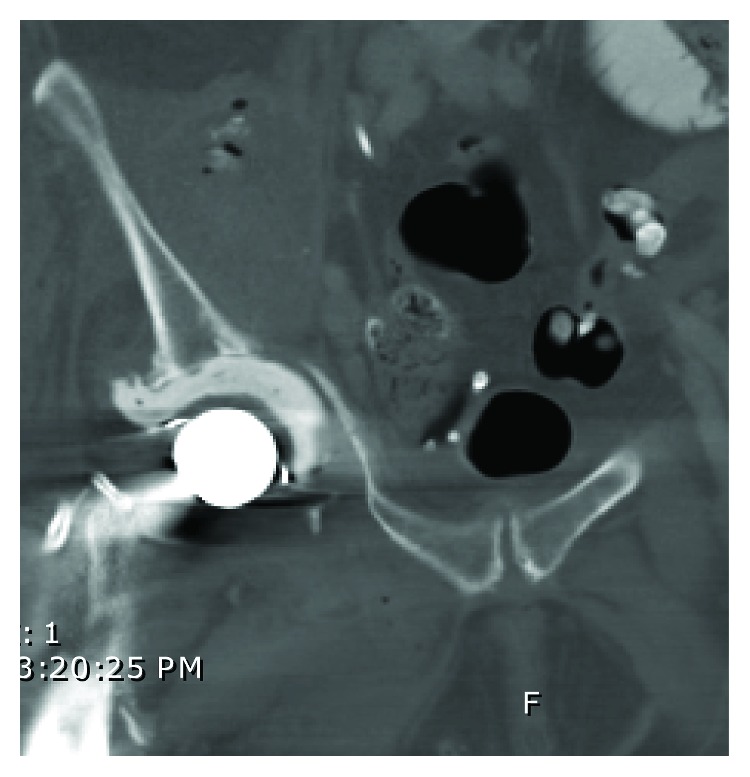
Stage 1: postoperative CT scan.

Temporary components were replanted with an antibiotic impregnated cement spacer system [13] (Figures 4 and 5). An antibiotic cement spacer with gentamicin was placed in the acetabulum defect, and a loosely fitted antibiotic-cemented stem was placed in the proximal femur.

**Figure 6:**
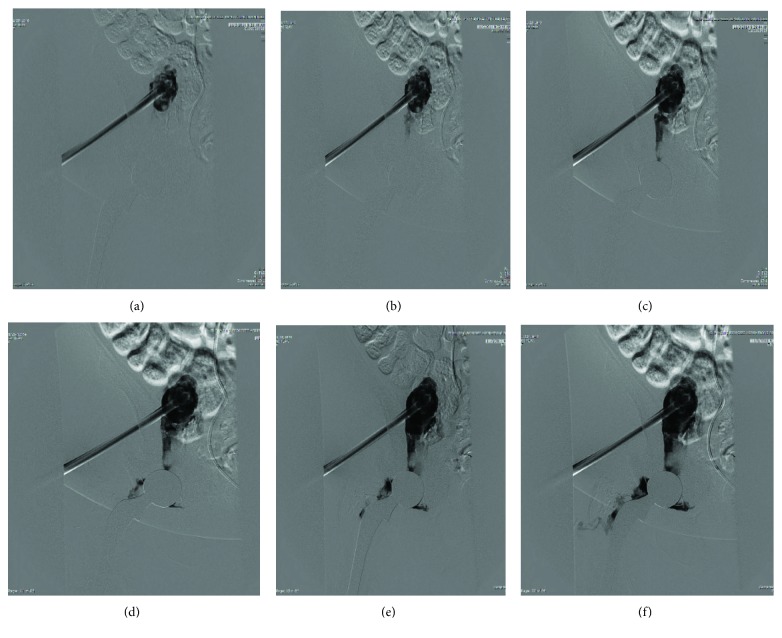
Flow of contrast dye tracking along the iliopsoas bursa into the hip joint.

The intrapelvic iliopsoas collection could not be fully debrided through the anterior approach, and IR was consulted for drain placement and serial debridements, which were conducted with a rotating basket Trerotola device over the course of the next four weeks. In Figure 6, contrast dye can be seen tracking from within the iliopsoas abscess into the hip joint.

**Figure 7:**
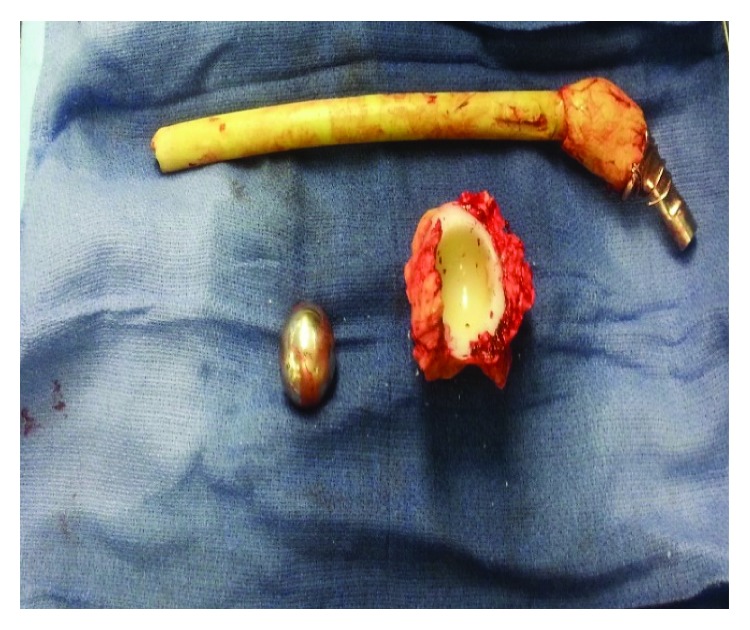
A custom-fitted temporary prosthesis.

**Figure 8:**
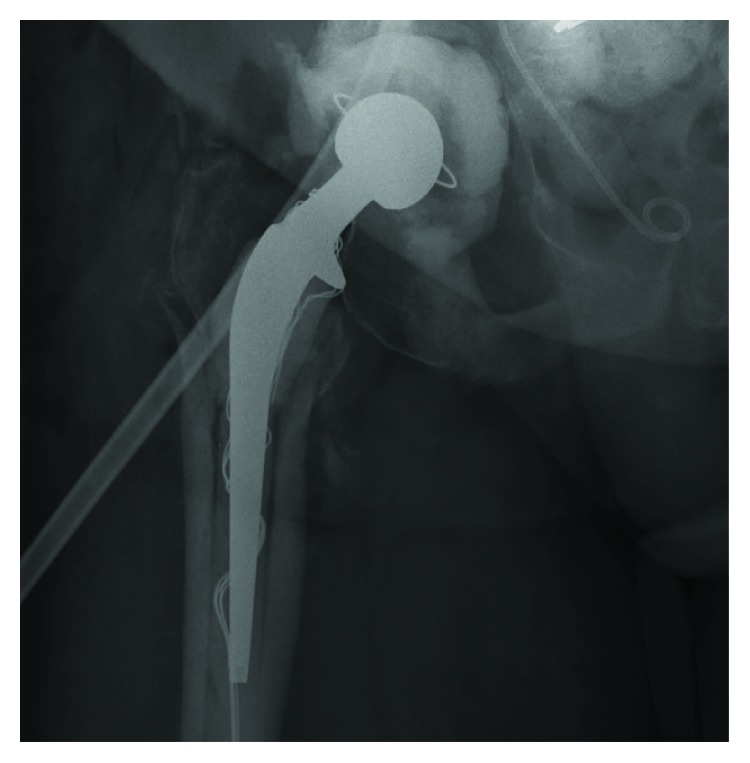
XR right hip. Stage 2: temporary prosthesis.

Four weeks following her explantation, the patient returned to the operating room for placement of a second temporary weight bearing custom-fitted prosthesis made from methyl methacrylate with gentamicin-impregnated antibiotics [13] (Figures 7 and 8). For the acetabulum, methyl methacrylate was molded in its doughy state into the cavities and deformities of the acetabulum, and a polyethylene acetabulum was pressed into the cement. The femur was reamed to size of a large diameter chest tube. A femoral stem and a reinforcing wire were cemented into the chest tube, and once the cement was hardened, the femoral stem encased in a solid cylinder of bone cement was removed from the chest tube and malleted into the proximal femur.

Postoperatively, the patient did well. She was made weight bearing as tolerated, ambulated with physical therapy and elected to delay placement of permanent components. She was eventually discharged to a short-term rehabilitation facility with a 6-week course of IV antibiotics.

**Figure 9:**
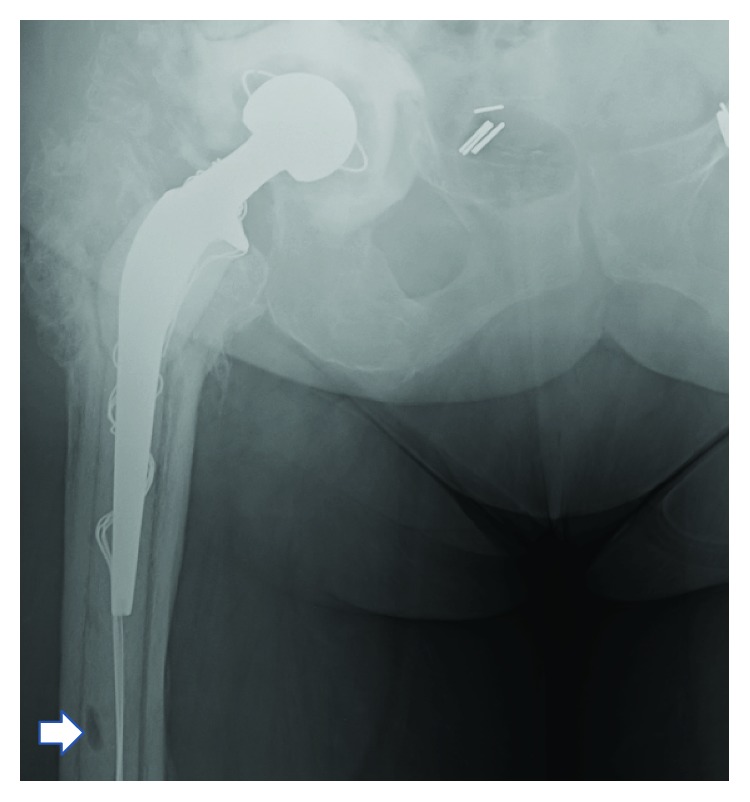
XR right hip. Radiolucencies around the femoral component (arrowhead).

**Figure 10:**
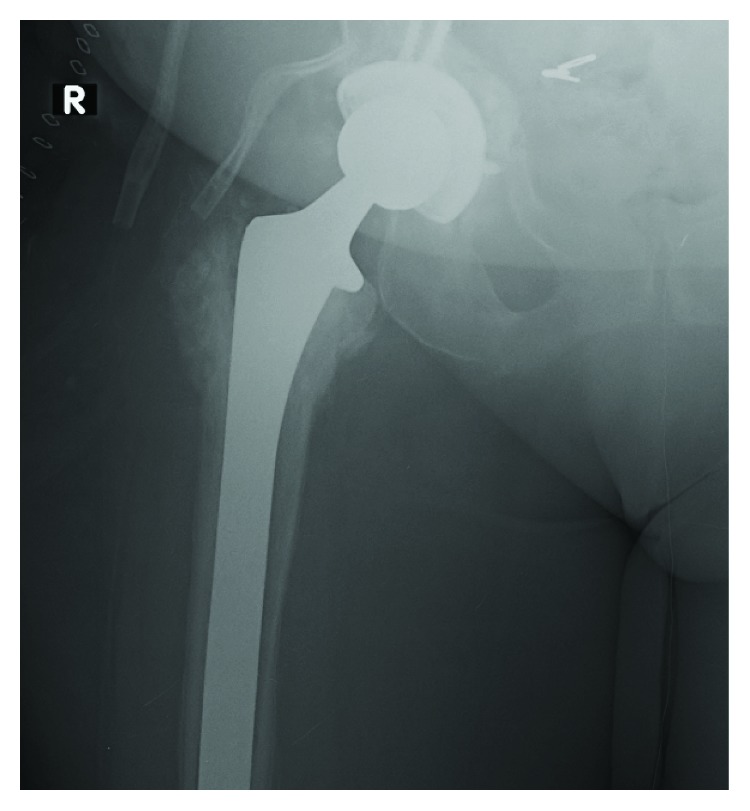
XR right hip. Long porous coated system.

Approximately 18 months later, she presented to our clinic complaining of hip and thigh pain with ambulation. She was followed by infectious disease as an outpatient with multiple hip aspirations which had negative cultures. X-rays revealed her temporary prosthesis to be stable, but with radiolucencies primarily around the femoral component (Figure 8). The patient was taken to the operating room, the temporary prosthesis was removed, and a long porous coated system was inserted (Figure 9). Intraoperative cultures grew vancomycin-resistant enterococcus (VRE), and the patient was eventually discharged to home with a 12-week course of daptomycin and outpatient physical therapy. Now, two years from her initial explantation, she continues to follow up monthly in our clinic and states that she is doing well. Her final construct is shown in Figure 10. She continues to have a moderate limp but ambulates without assistive devices. Her pain is much improved and she is no longer on chronic antibiotic suppression with no clinical signs or symptoms of recurrent infection.

## 3. Discussion

Our case demonstrates the unique consequence of spilled gallstones following LC. These stones can be spilled from manipulation during retraction, dissection, and removal of the gallbladder. In addition, abscesses are by far the most frequent complication [14]. They are usually located intraperitoneally (56%), either in the subhepatic region, abdominal wall (20%), thoracic (13%), and retroperitoneal space (11%) [15]. In a retrospective review, the median and mean times from LC to the first onset of symptoms were 3 and 5.5 months, respectively [16].

To our knowledge, this is the first reported case of dropped gallstones causing an iliopsoas abscess and a periprosthetic total hip infection. Chin et al. reports a case of a patient presenting 8 months after a laparoscopic cholecystectomy with a 6 × 4 cm firm mass superficial to the right hip joint in the iliopsoas bursa. The mass was excised, and an abscess cavity containing two large faceted gallstones was found [17]. There was no connection to the peritoneal cavity or any reported involvement of the hip joint or muscle.

It is our belief that the medial wall defect of the acetabulum caused by overmedialization of the patient's primary acetabular component provided the principle path of infection into the hip joint. However, it is also possible that the abscess tracked along the iliopsoas bursa into the hip joint. The fluoroscopic IR images demonstrate the flow of contrast from the intrapelvic abscess along the iliopsoas bursa into the hip joint. As previously discussed, this anatomical connection has been well documented in patients with tuberculous infections of the spine which communicates with the hip.

This case demonstrates the potential deleterious effects of spilled gallstones and subsequent abscess formation in patients with total hip prosthetics. This case is unique as an extensive review of the literature failed to show an association between dropped gallstones and iliopsoas abscesses or periprosthetic total hip infections. Surgeons taking care of patients with total hip prostheses with an overmedialized acetabulum component which has breached the medial cortex should be aware of this potential complication with LC. Finally, we would like to highlight the importance of custom-fitted temporary prosthesis. They have greater mechanical stability since they can be fashioned to deal with individual variations of destroyed bone or acetabular cavities. This in turn allows for better postoperative function, decreases the incidence of dislocation and minimizes pain [18]. In addition to these mechanical advantages, the type and dose of antibiotics mixed in with the methyl methacrylate can be tailored to address the specific bacteria encountered in any one case.
